# Prevalence and control of hypertension among a Community of Elderly Population in Changning District of shanghai: a cross-sectional study

**DOI:** 10.1186/s12877-017-0686-y

**Published:** 2017-12-28

**Authors:** Zhi-Qi Yang, Qi Zhao, Ping Jiang, Song-Bai Zheng, Biao Xu

**Affiliations:** 1 School of Public Health, Fudan University, Shanghai, China; 2 Key Laboratory of Public Health Safety (Fudan University), Ministry of Education, Shanghai, China; 3 Changning District Health and Family Planning Commission, Changning District, Shanghai, China; 4 Huadong Hospital Affiliated to Fudan University, Shanghai, China; 5 Department of Public Health Sciences (Global Health/IHCAR), Karolinska Institutet, Stockholm, Sweden; 6 Department of Epidemiology, School of Public Health, Fudan University, No.130 Dong-An Road, Shanghai, China

**Keywords:** Elderly population, Hypertension, Prevalence, Blood pressure control

## Abstract

**Background:**

Hypertension is considered a major public health challenge. It is the most important risk factor for cardiovascular disease and is a prominent risk for China’s elderly population. However, few studies have addressed the effect of blood pressure control on elderly hypertension patients in China. In response, this study aimed to investigate the prevalence and control of hypertension in the elderly population in Shanghai’s communities.

**Methods:**

A secondary data analysis based on a government-financed health check-up program for an elderly population aged 65 and older from 2012 to 2014 was conducted in a central district of Shanghai.

**Results:**

Of the 44,978 study participants, 20,305 (45.1%) were males and 24,673 (54.9%) were females. The participants’ median age was 72. Half of the participants were overweight or obese (BMI ≥ 24.0 kg/m^2^). The prevalence of hypertension was 59.9%. In the 18,032 participants without prior diagnosis of hypertension, 5530 (30.7%) had increased blood pressure. Among the 26,946 confirmed hypertension patients, the proportions of treatment and blood pressure control were 32.8% and 43.4% respectively. Multivariate analysis showed that the uncontrolled hypertension was significantly associated with older age, being overweight or obese, a lower level of education, an unbalanced dietary pattern, regular drinking and non-comorbidities.

**Conclusions:**

The prevalence of hypertension was high in China’s elderly population. The proportion of individuals who received treatment remained low, and blood pressure control was poor among hypertension patients. These results indicate that improvement of the ability to manage and control hypertension among urban elderly residents is urgently needed.

## Background

Globally, cardiovascular disease (CVD) became the leading cause of mortality in 2013 and was responsible for nearly one-third of all deaths [1]. In the meantime, hypertension, which is considered to be the most crucial risk factor for CVD, has caused half of cardiovascular mortality and morbidity and led to 9.4 million deaths per year [2]. Additionally, approximately 40% of adults who were 25 and above were diagnosed with hypertension around the world [2]. Hypertension also places a severe burden on China’s population.

According to the baseline survey from the nationwide China Health and Retirement Longitudinal Study (CHARLS), hypertension had a higher under-diagnosis rate among middle-aged and elderly Chinese [3]. The prevalence of hypertension among study respondents over 75 years was 58.0% for men and 62.1% for women [3]. Although hypertension has been considered a treatable condition, life-long intake of medication is required to control a patient’s blood pressure. In many regions of the world, especially in low and middle-income countries such as China, hypertension control remains a major health threat [4–6].

Population aging presents a tremendous challenge for China’s hypertension control. The United Nations estimated that individuals aged ≥60 constituted 16.2% of the total Chinese population in 2017, and they predicted the percentage would increase to 35.1% by 2050 [7]. As a large metropolitan city in China, the ageing population in Shanghai is increasingly serious, especially in central districts. Elderly people are at a higher risk of non-communicable diseases, hypertension and raised blood pressure (BP) [8]. In Shanghai, the prevalence of hypertension was 41.9% among individuals who were 34–74 years of age in the community population [9], and the prevalence was 59.4% among those aged ≥60 years [10]. Therefore, urgent action, optimal treatment approaches and proper public health strategies are needed to prevent and manage hypertension in the elderly population.

Despite the high prevalence of hypertension in China, there is a lack of updated epidemiological evidence for BP control among the elderly population, especially in cities suffering population ageing like Shanghai. Previous studies may also have defective small sample sizes, a failure to investigate the susceptible population, or an inability to examine hypertension controls [5, 11–15].

This cross-sectional study was conducted to look into the current situation of hypertension burden in elderly Chinese residents. The study was designed to investigate the BP distribution and treatment effect among both prevalent cases and new cases with abnormal BP. The study’s objectives included the following: first, to investigate the prevalence of hypertension among the community population of individuals who are 65 years and older in the Changning District, Shanghai. Second, to evaluate the effect of blood pressure control among elderly hypertension patients. Third, to explore the risk factors associated with suboptimal BP control.

## Methods

### Study participants and data source

This study was a secondary data analysis carried out in the Changning District, which is one of the central districts in Shanghai. The total coverage of the Changning District is 37.19 km^2^, including nine communities and one town. The Changning District has one of the highest proportion of elderly population in Shanghai. Among the district’s 590,000 permanent residents, 20.4% were 65 years or older with a life expectancy of 84 years in 2015 [16]. Data were obtained from a government-financed health information platform. The data included health archives, health check-up records and disease registrations with a solid information security system. After the approval of the research plan on elderly health, corresponding data were exported from the system without identification information. The study was based on the “Healthy Ageing” program. This program was conducted as part of the twelfth five-year plan (from 2011 to 2015) and ageing-related undertakings in Shanghai, China. The study was jointly conducted by the district Committee on Ageing and district Health & Family Planning Commission. Since 2012, the Healthy Ageing Program has provided health check-ups free of charge for elderly residents (aged 65 and older) living in the district to further implement health management among the elderly population. For this study, we extracted health check-up data and demographic information of all the elderly residents who received this free physical check-up during 2012–2014 from the District Health Information Center. A total of 44,978 subjects were included in the study population.

### Data collection

Information on social demographics, lifestyle, dietary habits, diseases and medication history were routinely recorded by trained health staff upon registration for the health check-up. Shanghai Municipal Center for Disease Control and Prevention started providing special spoons for residents to quantify salt intake in 2008. Drinking status was classified into three groups based on the frequency of alcohol consumption: never (never drinking), occasional (< once/week), regular (≥ once/week).

Blood pressure was measured using a calibrated electronic sphygmomanometer (Omron Corporation, HBP-1300, Kyoto, Japan). Participants were required to rest for at least 5 min before BP measurements. The left upper arm BP was measured 3 times in a sitting position with guidance from trained physicians. Measurements were considered to be unstable if the differences between the last two readings exceeded 5 mmHg and additional measurements were taken until the differences were lower than 5 mmHg. Systolic blood pressure (SBP) and diastolic blood pressure (DBP) were calculated as the averages of the last two readings of the three measurements.

### Definitions

Blood pressure was used to classify individuals into six groups: normal (SBP < 120 mmHg and DBP < 80 mmHg), prehypertension (SBP: 120–139 mmHg or DBP: 80–89 mmHg), stage one hypertension (SBP: 140–159 mmHg or DBP: 90–99 mmHg), stage two hypertension (SBP: 160–179 mmHg or DBP: 100–109 mmHg), stage three hypertension (SBP ≥ 180 mmHg or DBP ≥ 110 mmHg), and isolated systolic hypertension (SBP ≥ 140 mmHg and DBP < 90 mmHg). If any different classification of SBP and DBP seems applicable for the same person, the higher level shall prevail.

In this study, hypertension was defined as self-reported confirmed diagnosis of hypertension (medical certificates were checked by qualified physicians) and/or regular use of antihypertensive medication. The proportion of treatment was defined as the percentage of those who had taken antihypertensive medication, whereas the proportion of blood pressure control was defined as the percentage of individuals whose SBP and DBP were both lower than 140/90 mmHg among all the confirmed hypertension patients. The detection rate of raised blood pressure represented the proportion of participants who had SBP ≥ 140 mmHg or DBP ≥ 90 mmHg among individuals without a prior diagnosis of hypertension or use of antihypertensive medication. Comorbidity was defined as having hypertension and one or more co-existing conditions of these three non-communicable diseases (NCDs): diabetes, coronary heart disease (CHD), and stroke.

### Statistical analysis

A chi-square test and one-way ANOVA (for homoscedasticity) or Mann–Whitney U test (for heteroscedasticity) were used to examine differences in the categorical and continuous variables, respectively. Multivariate analysis through binary logistic regression was applied to search factors associated with uncontrolled BP. Age, gender, body mass index (BMI), education level, dietary pattern, salt intake, treatment for hypertension, and comorbidity of other NCDs were included as covariates. All statistical analyses were performed using SPSS software (version 19.0, Chicago, Illinois, USA). A two-sided *p* value <0.05 was considered statistically significant.

### Ethical considerations

This routine, record-based secondary data analysis was approved by the Ethic Committee in Huadong Hospital Affiliated to Fudan University (No. 2014 K004). Informed consent was exempt because there was no individual identification information in the analytical database. A unique code was given to each participant for individual differentiation. The government financed physical check-up was offered free of charge, and participation was voluntary. Data used in the analysis were secured and exported by information technology professionals in the district information center corresponding to the research plan.

## Results

### Demographic characteristics of participants

A total of 44,978 people aged 65 and older (age range: 65–112 years) took the health check-up provided by the “Healthy Ageing” program from 2012 to 2014. The median age of participants was 72 (interquartile range: 67–78 years), and 19.3% (8682/44,978) of them were 80 years or older. Participants who were 65–79 years old and over 80 years old accounted for 45.3% (36,296/80,100) and 23.2% (8682/37,400) of the whole population of their respective age groups in the district [16]. Overall, 45.1% (20,305/44978) of the participants were male and 50.1% (22,136/44,151) were overweight or obese. Additionally, 19.5% (8771/44,978), 18.0% (8112/44,978), and 7.1% (3178/44,978) of the participants reported a history of diabetes, coronary heart disease and stroke, respectively. Only 8.4% (3713/44,157) of the participants had no formal education, whereas 15.0% (6621/44,157) had a college degree or higher (Table 1).

**Table 1:** Demographic characteristics of participants

Demographic characteristics		Total		Hypertension		No confirmed hypertension		*p* value*
		(*N* = 44,978)		(*N* = 26,946)		(*N* = 18,032)		
		n	%	n	%	n	%	
Age (Years)	65–	17,348	38.6	9183	34.1	8165	45.3	<0.001
	70–	9105	20.2	5557	20.6	3548	19.7	
	75–	9843	21.9	6456	24	3387	18.8	
	80–	6347	14.1	4233	15.7	2114	11.7	
	85–	2335	5.2	1517	5.6	818	4.5	
Gender	Male	20,305	45.1	11,936	44.3	8369	46.4	<0.001
	Female	24,673	54.9	15,010	55.7	9663	53.6	
BMI (kg/m^2^)	0–	20,190	45.7	10,776	40.7	9414	53.2	<0.001
	18.5–	1825	4.1	726	2.7	1099	6.2	
	24.0–	16,688	37.8	10,864	41.1	5824	32.9	
	28.0–	5448	12.3	4080	15.4	1368	7.7	
Education	University	6621	15	3699	13.9	2922	16.6	<0.001
	Senior high school	11,074	25.1	6349	23.9	4725	26.8	
	Junior high school	14,704	33.3	8794	33.2	5910	33.5	
	Primary school	8045	18.2	5242	19.8	2803	15.9	
	No formal education	3713	8.4	2443	9.2	1270	7.2	
Dietary habit	Balanced	37,969	84.4	22,559	83.7	15,410	85.5	<0.001
	Meatarian	1645	3.7	1015	3.8	630	3.5	
	Vegetarian	5364	11.9	3372	12.5	1992	11	
Salt intake	≤ 6 mg/day	43,766	97.3	26,157	97.1	17,609	97.7	<0.001
	> 6 mg/day	1212	2.7	789	2.9	423	2.3	
Drinking	Never	29,121	70.8	17,838	72	11,283	69	<0.001
	Occasional	8299	20.2	4806	19.4	3493	21.4	
	Regular	3685	9	2118	8.6	1567	9.6	
Diabetes	No	36,207	80.5	20,090	74.6	16,117	89.4	<0.001
	Yas	8771	19.5	6856	25.4	1915	10.6	
CHD	No	36,866	82	20,790	77.2	16,076	89.2	<0.001
	Yas	8112	18	6156	22.8	1956	10.8	
Stroke	No	41,800	92.9	24,538	91.1	17,262	95.7	<0.001
	Yas	3178	7.1	2408	8.9	770	4.3	

*P values represented the comparisons between hypertension and not confirmed hypertension from chi-square tests

### Distribution of blood pressure

**Fig. 1:**
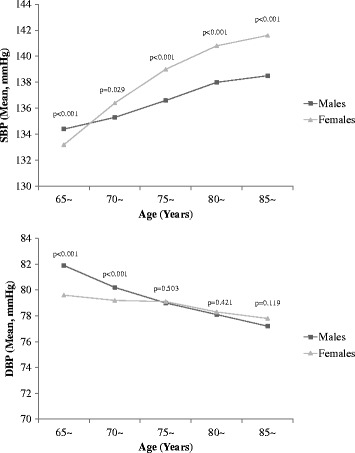
Mean systolic and diastolic blood pressure by age and sex among all participants. P values represented the differences of SBP or DBP between males and females within different age groups from Mann-Whitney U test due to heteroscedasticity

In Fig. 1, the mean SBP displayed an increasing trend with age for both sexes, and females had higher SBP than males at ages of 70 or older (*p* < 0.001). Meanwhile, an inverse association was observed between DBP and age. Mean levels of DBP were similar for males and females in the 75-year-old age group (*p* = 0.503); however, males had significantly higher DBP than females in age groups below 75 years (*p* < 0.001), whereas the females had a higher DBP in age groups over 75 years that was not significant (*p* = 0.130).

### Prevalence of hypertension and detection rate of raised blood pressure

Overall, 59.9% of participants (58.8% of males vs. 60.8% of females; *p* < 0.001) had hypertension. The prevalence increased significantly according to age (65–69: 52.9%, 70–74: 61.0%, 75–79: 65.6%, 80–84: 66.7%, 85-: 65.0%; *p* value for the liner trend test: <0.001). The prevalence of hypertension-diabetes, hypertension-CHD, and hypertension-stroke comorbidity was 15.2%, 13.7% and 5.4%, respectively.

Of the 18,032 participants who did not report a prior hypertension diagnosis or prior use of antihypertensive medication, 5530 (30.7%) were detected with raised blood pressure at SBP ≥ 140 mmHg or DBP ≥ 90 mmHg.

Based on the measured blood pressure, the proportions of participants with stage 1, 2, 3 and isolated systolic hypertension were 31.9%, 11.3%, 3.0% and 26.8%, respectively. In the meantime, only 11.8% of the participants were normotensive (Table 2).

**Table 2:** Grades of blood pressure among participants by non-communicable diseases history

Diseases	Number of Patients	%	SBP < 120 mmHg and DBP < 80 mmHg		SBP:120–139 mmHg or DBP:80–89 mmHg		SBP:140–159 mmHg or DBP:90–99 mmHg		SBP:160–179 mmHg or DBP:100–109 mmHg		SBP ≥ 180 mmHg or DBP ≥ 110 mmHg		SBP ≥ 140 mmHg and DBP < 90 mmHg	
			*n*	%	*n*	%	*n*	%	*n*	%	*n*	%	*n*	%
No Confirmed Hypertension	18,032	40.1	3627	20.1	8875	49.2	4281	23.7	1035	5.7	214	1.2	8641	32.1
Hypertension	26,946	59.9	1660	6.2	10,045	37.3	10,082	37.4	4027	14.9	1132	4.2	3428	19.0
Hypertension Only	13,801	30.7	842	6.1	5043	36.5	5241	38.0	2144	15.5	531	3.8	4230	30.6
Hypertension - Diabetes	6856	15.2	373	5.4	2612	38.1	2521	36.8	1002	14.6	348	5.1	2362	34.5
Hypertension - CHD	6156	13.7	414	6.7	2302	37.4	2252	36.6	901	14.6	287	4.7	2088	33.9
Hypertension - Stroke	2408	5.4	164	6.8	936	38.9	885	36.8	321	13.3	102	4.2	783	32.5
All Participants	44,978	100.0	5287	11.8	18,920	42.1	14,363	31.9	5062	11.3	1346	3.0	12,069	26.8

### Hypertension treatment and hypertension control

**Fig. 2:**
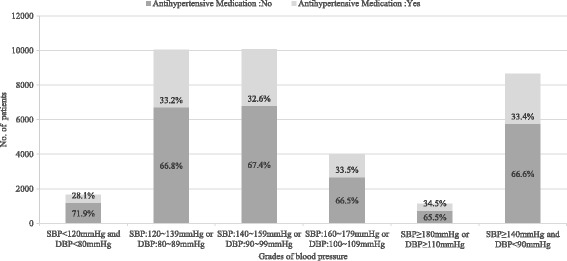
The proportion of antihypertensive treatment among hypertension patients with different blood pressure grades. The height of each stacked bar showed the absolute number of hypertension patients with the corresponding BP grade. The percentage in the light grey rectangle represented the proportion of those who had taken antihypertensive medication among hypertension patients with the corresponding BP grade; while the percentage in the dark grey rectangle represented the proportion of those who had not taken antihypertensive medication

Among all hypertension patients, only 32.8% (8829/26,946) were being treated. The treatment proportion was always below 35.0% in all blood pressure grades. This proportion did not vary substantially by sex (*p* = 0.416). Among individuals who received treatment for hypertension, 43.1% (3802/8829) achieved control of hypertension, and their average SBP and DBP were both lower than 140/90 mmHg. The proportion of hypertension control among all confirmed hypertension patients was 43.4% (11,705/26,946). The proportion of BP control was 59.4% (16,001/26,946) with the reference set as less than 150/90 mmHg according to the Eighth Joint National Committee (JNC-8) guidelines [17]. Only 6.2% (1660/26,946) of the hypertension patients had both SBP and DBP lower than 120/80 mmHg (Fig. 2).

### Factors associated with uncontrolled BP among hypertension patients

Unsatisfactory BP control was significantly associated with an increase in age. Additionally, the percentage of uncontrolled hypertension was slightly higher among females compared to males without statistical significance. Uncontrolled blood pressure was more common among individuals with a lower education level and a larger BMI. The dietary pattern and drinking frequency had a significant association with BP control, whereas salt intake did not. Hypertension patients who present comorbidity with other NCDs (diabetes, CHD and stroke) had a higher proportion of BP control than the ones without NCD comorbidity (Table 3).

**Table 3:** Associations between demographic and life-style factors and uncontrolled BP among elderly hypertension patients in Shanghai

Associated factors		Total	SBP > 140 mmHg or DBP > 90 mmHg		*p*	aOR (95% CI)^a^
			n	%		
Age (Years, Median & Interquartile range)			74(68–79)		<0.001	1.01 (1.01–1.02)
Sex	Male	11,936	6692	56.1		Ref.
	Female	15,010	8549	57.0	0.531	1.02 (0.96–1.08)
BMI(kg/m^2^)	0–	726	336	46.3	0.001	0.77 (0.65–0.90)
	18.5–	10,776	5648	52.4		Ref.
	24.0–	10,864	6366	58.6	<0.001	1.29 (1.22–1.37)
	28.0–	4080	2612	64.0	<0.001	1.61 (1.48–1.74)
Education	University	3699	1928	52.1		Ref.
	Senior high school	6349	3516	55.4	0.003	1.14 (1.04–1.24)
	Junior high school	8794	5013	57.0	<0.001	1.19 (1.09–1.29)
	Primary school	5242	3095	59.0	<0.001	1.26 (1.14–1.38)
	No formal education	2443	1457	59.6	<0.001	1.25 (1.11–1.41)
Dietary pattern	Balanced	22,559	12,690	56.3		Ref.
	Meatarian	1015	627	61.8	0.003	1.23 (1.07–1.40)
	Vegetarian	3372	1924	57.1	0.210	1.05 (0.97–1.13)
Salt intake	≤ 6 mg/day	26,157	14,811	56.6		Ref.
	> 6 mg/day	789	430	54.5	0.108	0.89 (0.76–1.03)
Drinking	Never	17,838	10,002	56.1		Ref.
	Occasional	4806	2676	55.7	0.141	1.06 (0.98–1.13)
	Regular	2118	1262	59.6	0.001	1.19 (1.07–1.31)
Treatment^b^	Yes	8829	5027	56.9		Ref.
	No	18,117	10,214	56.4	0.239	0.97 (0.92–1.02)
Comorbidity^c^	Hypertension only	14,595	8335	57.1	0.001	Ref.
	2 NCDs	9555	5346	55.9	0.002	0.91 (0.86–0.97)
	≥ 3 NCDs	2796	1560	55.8	0.004	0.88 (0.80–0.96)

^a^Adjusted odds ratio and 95% confidence interval

^b^Treatment was defined as taking anti-hypertensive medication

^c^Co-morbidity was defined as having hypertension and ≥1 of the following three diseases: diabetes, coronary heart disease, and stroke

## Discussion

To the best of our knowledge, this study was one of the largest studies in China seeking to discover the prevalence and control hypertension among the elderly population. Overall, 38.3% (44,978/117,500) in the base population in Changning district were enrolled. This study provided new evidence signifying the current burden of hypertension and the critical situation for uncontrolled hypertension among the elderly population in central Shanghai, which is city in China with the largest number of elderly individuals. Furthermore, because the studied population was composed of a younger population compared to the base population, the disease burden might be underestimated.

This study showed that approximately 60% of the elderly residents in Changning had hypertension, and this crude prevalence of hypertension increased substantially with age. Meanwhile, the prevalence was higher in elderly females than males, which was a factor that was not observed in previous studies [11]. Hypertension prevalence in the present study was comparable to reports of previous surveys in China [11, 18]. Nonetheless, the prevalence found in this study was slightly lower than the study results from some high-income countries such as the United States [19]. A similar issue was also recognized in hypertension control. According to this study, only one-third of the hypertension patients aged 65 years and older had taken hypertensive medication to provide BP control. The proportion of treatment was much lower compared to other studies in China [10, 20], which might be because Chinese studies usually took the hypertension patients under a specific NCD control program as the denominators when reporting treatment coverage, whereas the denominator for this study was all hypertension patients regardless of their participation in a hypertension management program. The treatment coverage is unsatisfactory compared to high- and upper middle-income countries [11]. Although the proportion of controlled hypertension (43.4%) in this study was relatively higher compared to other developing countries [11], the control effect of hypertension was still unsatisfactory compared to many developed regions, such as Europe and the United States [21, 22]. Therefore, treatment management for elderly hypertension patients would be a tremendous challenge to the Shanghai health authority and health system.

Old age, being overweight and obesity are traditional risk factors for uncontrolled hypertension [20]. In the meantime, the results of the present study provided additional support for that evidence. A recent study on elderly Chinese individuals aged 80 years or older estimated a hypertension prevalence of 75.3%, while 45.5% of hypertension was treated, and only 18.1% of hypertensive participants were controlled [15]. It is revealed that the importance of hypertension prevention and management for China’s elderly population would increase sharply with ageing. In the meantime, obesity also provides predictive information on atherosclerotic CVDs [23]. Previous studies indicated that abdominal obesity and visceral obesity were associated with increased hypertension prevalence among elderly population [15, 24]. Therefore, non-hypertensive elders can prevent hypertension through lifestyle modification and the reinforcement of daily exercise. These methods are also beneficial for the optimal control of BP among elderly patients. As indicated in other reports [25], elderly patients with less education showed less efficient hypertension control. Education is an important tool that leads to a higher socioeconomic status and healthier lifestyle habits, which could both strengthen and improve levels of awareness, treatment and control of hypertension. It has been shown that lack of knowledge hinders medication adherence, especially for an elderly population [26]. Results from multivariate analysis also indicated that the BP control among males was slightly better than BP control among females, although the difference was not statistically significant. Some studies conducted in other countries, such as Japan, Poland and the United States, reported higher hypertension prevalence and worse BP control among elderly females than elderly males [27–29]. Further population-based studies are still needed to confirm whether an association exists between sex and BP control among the elderly population. On the other hand, more attention should be given to the effects of BP control among female patients because high blood pressure has a greater impact on cardiovascular risk in females than in males, and females after menopause are especially vulnerable [30, 31].

Previous studies suggested that a balanced diet plays an important role in hypertension prevention and control [32, 33]. Based on the present study, a dietary pattern with high consumption of meat was associated with uncontrolled hypertension. However, a null association between vegetable consumption and BP was observed, which was consistent with several other observational studies [34, 35]. The Dietary Approaches to Stop Hypertension (DASH) intervention study has shown that a diet rich in fruits, vegetables, and low-fat dairy products as well as low saturated fat can substantially lower both SBP and DBP [36]. Hypertension patients who drank regularly were less likely to have their BP under control. A Korean study discovered the adverse effects of drinking was significant for uncontrolled BP both at nighttime and over 24 h [37]. In fact, heavy drinking itself can be associated with resistant hypertension [38]. In addition, a previous meta-analysis showed that high salt intake was highly prevalent in low- and middle-income countries, and excessive use of salt had a greater impact on hypertension burden in urban regions than rural regions [39]. However, a significant relationship between salt intake and BP control was not observed in this study, which likely occurred due to the generally light cooking style in Shanghai.

Optimal BP control in elderly hypertension patients with comorbidities was achieved with less difficulty than in patients without comorbidities. Similar results were found in the Oslo Health Study 2000–2001 that showed the presence of diabetes or CVD was independently associated with better BP control [40]. In comorbidity situations, diabetes and cardio-cerebral vascular diseases can be aggravated by high or uncontrolled BP. Thus, patients with comorbid conditions are more likely to have frequent hospitalizations and adverse treatment outcomes. Therefore, BP control should be considered the priority for patients with comorbidities, especially for elderly patients. Indeed, there was a null association between antihypertensive treatment and BP control because only one-third of hypertension patients were treated with antihypertensive medication. However, an appropriate combination of antihypertensive medication treatment is still crucial for optimal BP control in both central regions and suburban or rural regions that have patients with less education and limited qualified medical resources [20].

Nevertheless, this study has limitations. First, it is a secondary analysis of cross-sectional data, so this study could not establish a cause-effect relationship between postulated factors and uncontrolled BP. Second, the study is missing some information, such as the exact date of diagnosis, duration of treatment, and social-economic status, such as family income level, that might be associated with hypertension control. Third, participants in this study received the health check-up on a volunteer basis and they might have younger ages and healthier physical conditions than the actual base population [16]. As a result, generalization of these findings from this study to other elderly populations in China should be handled with caution.

## Conclusions

Among elderly residents (aged 65 and above) in central Shanghai, China, the prevalence of hypertension was high, yet the control of hypertension was unsatisfactory. Hypertension control is an essential precondition for reducing the impact, complications and deaths from cardiovascular diseases. This study yielded important information to improve our understanding of the hypertension burden. The study also contributed to clarifying the factors that have hindered hypertension control in this elderly population. Development of strategies that improve the management and control of hypertension among urban elderly residents, especially among hypertension patients without other NCDs are needed to respond to the urgent needs for disease control and comorbidity prevention.

## Abbreviations

BMI: Body mass index
BP: Blood pressure
CHARLS: China Health and Retirement Longitudinal Study
CHD: Coronary heart disease
CVD: Cardiovascular disease
DASH: Dietary Approaches to Stop Hypertension
DBP: Diastolic blood pressure
JNC-8: The Eighth Joint National Committee
NCDs: Non-communicable diseases
SBP: Systolic blood pressure
